# Zika: A scourge in urban slums

**DOI:** 10.1371/journal.pntd.0005287

**Published:** 2017-03-23

**Authors:** Robert E. Snyder, Claire E. Boone, Claudete A. Araújo Cardoso, Fabio Aguiar-Alves, Felipe P. G. Neves, Lee W. Riley

**Affiliations:** 1 Division of Epidemiology, School of Public Health, University of California Berkeley, Berkeley, California, United States of America; 2 Maternal and Child Department, School of Medicine, Universidade Federal Fluminense, Niterói, Rio de Janeiro, Brazil; 3 Pathology Program, Laboratório Universitário Rodolpho Albino, Universidade Federal Fluminense, Niterói, Rio de Janeiro, Brazil; 4 Department of Microbiology and Parasitology, Biomedical Institute, Universidade Federal Fluminense, Niterói, Rio de Janeiro, Brazil; 5 Division of Infectious Diseases and Vaccinology, School of Public Health, University of California Berkeley, Berkeley, California, United States of America; Yale University Yale School of Public Health, UNITED STATES

In December 2014, during the height of the Ebola virus disease (EVD) epidemic in West Africa, we wrote, “EVD is only the beginning and only one disease; even if we are to control the current epidemic, the future introduction of this and other highly contagious and virulent microbes to and from global slums is inevitable” [1].

Fast forward to late 2015: another epidemic of another virus—Zika—together with its major neurological complication—microcephaly—explodes in Brazil. Again, as with the EVD epidemic, the world’s attention is focused on issues such as where the virus originated, the need to control vectors, how quickly we can develop a vaccine, and how long the epidemic will continue. While these are important discussions to have, they are irrelevant if the world does not recognize and address a crucial reason why these explosive epidemics continue to occur in the first place: the world must talk about urban slums.

Zika is, and will continue to be, a disease of the urban poor. Slum-defining characteristics—poor water and sanitation infrastructure, crowding, and poor structural quality of housing—offer ample opportunities for mosquitoes to breed and spread the Zika virus. It was recently estimated that 1.6 million childbearing women and 93 million people will be infected in the Americas’ first epidemic wave [2]. From this reservoir of infections, the world is witnessing the largest epidemic ever of a congenital complication—microcephaly. Except for sporadic reports, largely absent from discussion is the fact that the greatest proportion of Zika infections and its complications have occurred, and will continue to occur, among residents of the large, densely packed informal human settlements of Latin America and the Caribbean.

The *Aedes aegypti* mosquito—the species that most commonly transmits Zika—will, on average, travel a mere 100 meters in its lifetime [3]. In the densely populated *favelas*, or slums of Brazil and elsewhere in the region, a single 100 square meter space could contain more than 100 housing structures, 2–3 stories high, with a resident population upwards of 1,000. These residents can be infected multiple times by mosquitoes circulating in such neighborhoods. Wealthy residents of high-rise apartment buildings with screened windows, air conditioning, and regular spraying of insecticides, even if located adjacent to these *favelas*, are less likely to be exposed to such mosquitoes.

The burden of symptomatic dengue, another mosquito-borne disease, has been shown to be greater among the neighborhoods with the lowest socioeconomic status within a slum [4]. In a study of successive dengue epidemics in Rio de Janeiro that occurred in 2007 and 2008, Rosa-Freitas et. al demonstrated that dengue incidence positively correlated with neighborhoods (*bairros*) with a larger proportion of their land area composed of urban slums [5]. In Singapore in the late 1960s, the fraction of houses that tested positive for *Aedes* mosquito breeding was highest in the city's slums and in those with greatest population density. [6].

Athletes and spectators who attended the 2016 Summer Olympic Games returned to their home countries with no subsequently reported Zika infections [7]. As the previous epidemic wave wanes in Brazil, it is clear that the ominous predictions about Zika virus’ global spread due to the Olympic and Paralympic Games were little more than demagoguery. Epidemiologic evidence always suggested that the epidemic would taper off well before the event [8]. Even a cursory analysis of other mosquito-borne diseases in Brazil carried by *A*. *aegypti*, such as dengue virus, reveals that peak incidence occurs well before August, during the hottest and wettest months, and then plummets in the winter (Fig 1) [9–12]. Indeed, even the Brazilian Ministry of Health’s most recent *Epidemiologic Bulletin* reported that there were zero new cases in Rio de Janeiro between epidemiological weeks 32 and 37 (early August to September), the most recent dates for which data were available at the time of publication [12]. Meanwhile, as of December, 2016, the United States has seen 185 cases of local mosquito transmission of Zika virus in two separate states (Texas and Florida), and not coincidentally, many of these cases occurred during the Northern Hemisphere’s warmest summer months [13]. While the global spotlight inaccurately highlighted the risk of Zika among international travelers and athletes during the Games, the ongoing weekly occurrence of microcephalic babies born to mothers who were exposed to Zika months ago in Rio’s sprawling slums is seemingly invisible.

**Fig 1:**
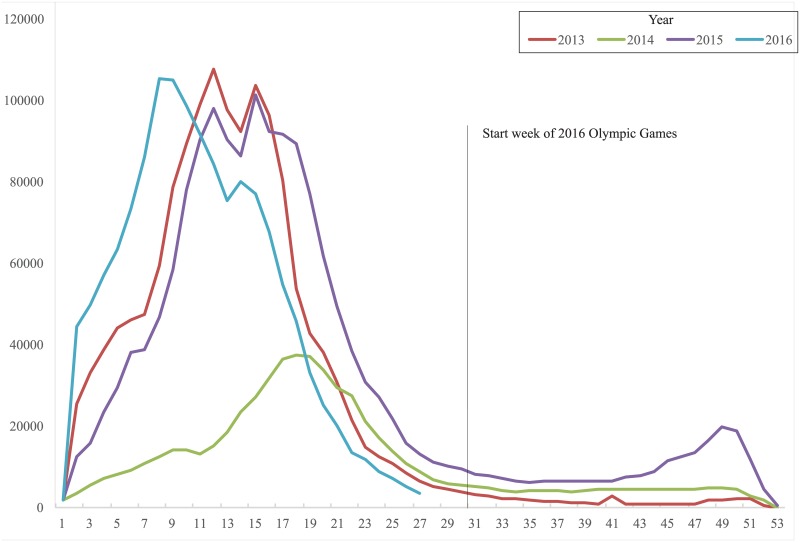
Dengue incidence per 100,000 population in Brazil, by epidemiologic week when symptoms began in 2013, 2014, 2015, and 2016. Created by extracting data from *Epidemiologic Bulletins* using WebPlotDigitizer 3.1 (http://arohatgi.info/WebPlotDigitizer/citation.html).

In the developed world, despite the low predicted and observed risk of importation and subsequent ongoing transmission of Zika virus due to the 2016 Olympic and Paralympic Games [14], we continue to focus on the implications of the disease among low-risk populations, ignoring the implications for disadvantaged populations. Predictive models suggest that the ongoing epidemic may “run out of steam” after two or three more seasons [15]. Unfortunately, none of these models consider how urban slums and their highly mobile populations affect the disease’s dynamics. In Brazil, dengue virus—a virus genetically similar to Zika—goes through seasonal epidemics with variations in annual incidence and disease severity [16], and there is nothing to suggest that Zika virus will not behave similarly. While it is true that so far there is only one major viral clade of Zika circulating in Latin America, this RNA virus is mutating rapidly, likely driven by the selective pressure of the hundreds of thousands of transmissions facilitated by the region’s densely packed slum populations. It is nothing more than human hubris to think that mosquitoes can or will be controlled in these slums. It is another act of hubris to think that a new vaccine, even if developed and shown to be effective—in an animal model or phase II clinical trial carried out in a highly controlled environment among healthy volunteers in wealthy countries—will successfully control this, or future epidemics of this virus that ravage urban slums. Of course, that is not to say that a well-designed vaccine could not reduce risk of infection in pregnant women and its neurological complications in children, assuming that the poverty and disenfranchisement of slum dwellers will not prevent them from being among the last to receive such a vaccine despite being at greatest risk.

The energy, media attention, and research resources revolving around the Zika virus epidemic must be harnessed and used to bring the largely ignored urban slum populations of megacities around the world into the global spotlight. Otherwise, we will continue to have the same inconsequential discussions until the next deadly epidemic is sparked in slums.

## References

[1] Snyder RE, Marlow MA, Riley LW. Ebola in urban slums: the elephant in the room. Lancet Glob Health. 2014;2(12): e685 10.1016/S2214-109X(14)70339-0 25433618 PMC5004591

[2] Perkins TA, Siraj AS, Ruktanonchai CW, Kraemer MUG, Tatem AJ. Model-based projections of Zika virus infections in childbearing women in the Americas. Nat Microbiol. 2016;1: 16126 10.1038/nmicrobiol.2016.126 27562260

[3] Hemme RR, Thomas CL, Chadee DD, Severson DW. Influence of Urban Landscapes on Population Dynamics in a Short-Distance Migrant Mosquito: Evidence for the Dengue Vector Aedes aegypti. PLoS Negl Trop Dis. 2010;4(3): e634–e639. 10.1371/journal.pntd.0000634 20300516 PMC2838782

[4] Kikuti M, Cunha GM, Paploski IAD, et al Spatial Distribution of Dengue in a Brazilian Urban Slum Setting: Role of Socioeconomic Gradient in Disease Risk. PLoS Negl Trop Dis. 9(7): e0003937 10.1371/journal.pntd.0003937 26196686 PMC4510880

[5] Rosa-Freitas MG, Tsouris P, Reis IC, et al Dengue and Land Cover Heterogeneity in Rio de Janeiro. Oecologia Australis. 2010;14(3): 641–667.

[6] Chan YC, Ho BC, Chan KL. Aedes aegypti (L.) and Aedes albopictus (Skuse) in Singapore City: 5. Observations in relation to dengue haemorrhagic fever. Bull. World Health Organ. 1971;44(5):651–657. 5316749 PMC2427849

[7] Payne, M. (2016 September 3) No new cases of Zika connected to the Olympics, WHO says. The Washington Post. https://www.washingtonpost.com/news/early-lead/wp/2016/09/03/no-new-cases-of-zika-connected-to-the-olympics-who-says/?utm_term=.47af53f80de8.

[8] San Martin JL, Brathwaite O, Zambrano B, et al The Epidemiology of Dengue in the Americas Over the Last Three Decades: A Worrisome Reality. Am J Trop Med Hyg. 2010;82(1): 128–135. 10.4269/ajtmh.2010.09-0346 20065008 PMC2803522

[9] Secretaria de Vigilância em Saúde − Ministério da Saúde. SUS- Portal de Saúde: Dengue, Chikingunya e Zika: Situação Epidemiológica / Dados. http://portalsaude.saude.gov.br/index.php/situacao-epidemiologica-dados-dengue (accessed Sep 8, 2016).

[10] Secretaria de Vigilância em Saúde − Ministério da Saúde. Monitoramento dos casos de dengue, e febre de chikungunya até a Semana Epidemiológica (SE) 53 de 2014. Boletim Epidemiológico. 2015;46(3): 1–10.

[11] Secretaria de Vigilância em Saúde − Ministério da Saúde. Monitoramento dos casos de dengue, febre de chikungunya e febre pelo vírus Zika até a Semana Epidemiológica 52, 2015. Boletim Epidemiológico. 2015;47(3): 1–10.

[12] Secretaria de Vigilância em Saúde − Ministério da Saúde. Monitoramento dos casos de dengue, febre de chikungunya e febre pelo vírus Zika até a Semana Epidemiológica 27 37, 2016. Boletim Epidemiológico. 2016;47(31): 1–10.

[13] Centers for Disease Control and Prevention. Zika Virus: Case Counts in the US [Internet]. 2016 [accessed 7 December 2016]. http://www.cdc.gov/zika/geo/united-states.html.

[14] Grills A, Morrison S, Nelson B, Miniota J, Watts A, Cetron MS. Projected Zika Virus Importation and Subsequent Ongoing Transmission after Travel to the 2016 Olympic and Paralympic Games—Country-Specific Assessment, July 2016. MMWR Morb Mortal Wkly Rep. 2016;65(28): 711–715. 10.15585/mmwr.mm6528e1 27442184

[15] Ferguson NM, Cucunubá Z, Dorigatti I, et al Countering the Zika epidemic in Latin America. Science. 2016;353(6297): 353–354. 10.1126/science.aag0219 27417493 PMC5475255

[16] Teixeira MG, Siqueira JB, Ferreira GLC, Bricks L, Joint G. Epidemiological Trends of Dengue Disease in Brazil (2000–2010): A Systematic Literature Search and Analysis. Unnasch TR, ed. PLoS Negl Trop Dis. 2013;7(12): e2520–13. 10.1371/journal.pntd.0002520 24386496 PMC3871634

